# Assessment of the genetic diversity of the Tunisian citrus rootstock germplasm

**DOI:** 10.1186/1471-2156-13-16

**Published:** 2012-03-19

**Authors:** Hager Snoussi, Marie-France Duval, Andres Garcia-Lor, Zina Belfalah, Yann Froelicher, Ange-Marie Risterucci, Xavier Perrier, Jean-Pierre Jacquemoud-Collet, Luis Navarro, Moncef Harrabi, Patrick Ollitrault

**Affiliations:** 1 Horticultural Laboratory, Tunisian National Agronomic Research Institute (INRAT), Rue Hedi Karray, 2049 Ariana, Tunisia; 2 Department BIOS, TGU AGAP, International Center for of Agricultural Research for Development (CIRAD), Avenue Agropolis, TA A-75/02, F-34398 Montpellier Cedex 5, France; 3 Centro de Protección Vegetal y Biotecnología, Instituto Valenciano de Investigaciones Agrarias (IVIA), Crta Moncada-Naquera 5, Valencia 46113, Spain; 4 Agronomy and Plant Biotechnology Department (INAT), Tunisian National Agronomic Institute (INAT), 43 Avenue Charles Nicolle, 1082 Tunis, Mahrajène, Tunisia

## Abstract

**Background:**

Citrus represents a substantial income for farmers in the Mediterranean Basin. However, the Mediterranean citrus industry faces increasing biotic and abiotic constraints. Therefore the breeding and selection of new rootstocks are now of the utmost importance. In Tunisia, in addition to sour orange, the most widespread traditional rootstock of the Mediterranean area, other citrus rootstocks and well adapted to local environmental conditions, are traditionally used and should be important genetic resources for breeding. To characterize the diversity of Tunisian citrus rootstocks, two hundred and one local accessions belonging to four facultative apomictic species (*C. aurantium*, sour orange; *C. sinensis*, orange; *C. limon*, lemon; and *C. aurantifolia*, lime) were collected and genotyped using 20 nuclear SSR markers and four indel mitochondrial markers. Multi-locus genotypes (MLGs) were compared to references from French and Spanish collections.

**Results:**

The differentiation of the four varietal groups was well-marked. The groups displayed a relatively high allelic diversity, primarily due to very high heterozygosity. Sixteen distinct MLGs were identified. Ten of these were noted in sour oranges. However, the majority of the analysed sour orange accessions corresponded with only two MLGs, differentiated by a single allele, likely due to a mutation. The most frequent MLG is shared with the reference sour oranges. No polymorphism was found within the sweet orange group. Two MLGs, differentiated by a single locus, were noted in lemon. The predominant MLG was shared with the reference lemons. Limes were represented by three genotypes. Two corresponded to the 'Mexican lime' and 'limonette de Marrakech' references. The MLG of 'Chiiri' lime was unique.

**Conclusions:**

The Tunisian citrus rootstock genetic diversity is predominantly due to high heterozygosity and differentiation between the four varietal groups. The phenotypic diversity within the varietal groups has resulted from multiple introductions, somatic mutations and rare sexual recombination events. Finally, this diversity study enabled the identification of a core sample of accessions for further physiological and agronomical evaluations. These core accessions will be integrated into citrus rootstock breeding programs for the Mediterranean Basin.

## Background

Worldwide production of citrus in 2009 reached greater than 120 million tons [1], making citrus the leading cultivated tree crop in the world. Oranges represent the majority of citrus production (54% in 2009) with over 67 million tons in 2009 [1]. The other significant cultivated citrus are mandarins, lemons and grapefruits. The citrus production of the Mediterranean Basin is second only to Brazil. Cultivars are vegetatively propagated by bud-grafting onto seedling rootstocks. This ensures tree uniformity, early tree production, and tolerance to pathogens including *Phytophthora *sp., parasitic nematodes and viruses. Rootstocks are also significant in the adaptation of the tree to several abiotic constraints affecting the Mediterranean citriculture. These include water resource scarcity and soil salinity. Citrus rootstocks are generally apomictic and seed-propagated. Therefore, both scions and rootstocks are clonally propagated. Sour orange (C. *aurantium *L.), one of the most important citrus rootstocks in the world, is still the predominant rootstock on the southern edge of the Mediterranean Basin. Sour orange is tolerant to limestone, alkalinity and salinity and is resistant to *Phytophthora *sp. Furthermore, sour orange is widely compatible with scion varieties and confers good fruit quality. Unfortunately, combining sour orange with commercial citrus varieties results in trees that are sensitive to Citrus Tristeza Virus (CTV), causing the rapid decline and death of the trees grafted on sour orange rootstocks. As CTV spreads throughout the Mediterranean basin, citrus production on sour orange rootstock will soon be uneconomic. On the other hand, rootstocks selected for their resistance to CTV are not well adapted to other local constraints [2]. Thus, there is an urgent need to diversify and select new citrus rootstocks exhibiting CTV resistance and adaptation to the regional abiotic stresses.

The most widely accepted taxonomic systems for *Citrus *are those of Swingle and Reece [3] and Tanaka [4], who recognized 16 and 162 species, respectively. Later, phylogenetic analysis by Scora [5] and Barrett and Rhodes [6] indicated only three true species within the cultivated citrus, i.e., *C. medica *L. (citron), *C. reticulata *Blanco (mandarin) and *C. maxima *(Burm.) Merr. (pummelo). Recent molecular studies have confirmed the central role of these three taxa [7-15] and concluded in favour of a fourth additional ancestral taxon, *C. micrantha *Wester [7,8]. The secondary species, *C. aurantium *L. (sour orange), *C. limon *(L.) Burm.f. (lemon), *C. aurantifolia *(Christm.) Swing. (lime), *C. sinensis *(L.) Osb. (sweet orange) and *C. paradisi *Macfad. (grapefruit), were derived from hybridization amongst the true species [7,9,10,16]. Despite significant phenotypical differentiation, all *Citrus *species and several related genera are sexually compatible [17,18] and can be considered part of the same biological species. Most citrus species are characterized by facultative apomixis resulting from adventitious nucellar embryogenesis [19]. This has deeply affected the intra- and inter-specific evolution of cultivated citrus. The total sexual compatibility within the genus permitted the occurrence of numerous inter-specific hybrids, which evolved through vegetative propagation due to their facultative apomixis. This led taxonomists to consider clonally propagated families of inter-specific origin as new species. As a result, *Citrus *taxonomy and systematics remain controversial [5]. Citrus was domesticated in Southeast Asia, notably East India, North Burma and Southwest China, and then spread to other continents [5,20]. Citrus introduction to Tunisia likely occurred during the 10^th ^century, and the citrus industry was established in the beginning of the 20^th ^century. Since 1934, export trade has undergone great expansion, and producers began producing the 'Maltaise demi-sanguine' orange. Today, citrus orchards occupy approximately 20,400 hectares, with citrus production oscillating between 210,000 and 300,000 tons during the last decade. Sour orange is still the most widely used traditional rootstock of Tunisian citriculture. Despite clonal propagation, the morphological variation exhibited by local accessions of sour orange is remarkable. Additional rootstock varieties and non-grafted citrus trees are still used, particularly in the oasis areas, and these trees are related to *C. sinensis, C. limon *and *C. aurantifolia*. The traditional Tunisian rootstocks are well adapted to the adverse regional soils and climatic conditions. Consequently, these rootstocks constitute a very important germplasm to be employed as an abiotic stress tolerance source for future rootstock breeding projects. Therefore, the activities of investigation, collection, preservation and characterization of this germplasm are a priority. The assessment of genetic diversity within the rootstock germplasm is a prerequisite to the optimization of its management at the national and regional level.

Simple sequence repeat (SSR) markers have gained considerable importance in plant genetics due to their many desirable genetic attributes, including high polymorphism, wide genomic distribution, co-dominant inheritance and reproducibility allowing networking activities, multi-allelic nature and chromosome-specific location. In citrus, nuclear SSR development from genomic libraries [21-24], ESTs [25,26] and BACend sequences [14] has been important during the last decade. These markers have proved to be very useful for the evaluation of genetic diversity in citrus [13,15] and for the analysis of the sexual or apomictic origin of plant seedlings [27-29].

The main objective of this study was to assess the genetic diversity of Tunisian citrus rootstock germplasm. Specifically, the following questions were addressed: (i) What are the extent and the structure of the Tunisian rootstocks genetic diversity? (ii) What are the origin and the extent of the genetic diversity within facultative apomictic species? (iii) How is the Tunisian germplasm diversity related to reference genotypes of the same varietal groups of certified Citrus collections? For this purpose, 201 individual from different regions of Tunisia were analysed, and a set of reference cultivars from Spanish and French germplasm banks were genotyped for 20 nuclear SSR markers and four Indels mitochondrial markers.

## Results

### Genetic diversity of Tunisian germplasm

#### Allelic diversity and its organization

All 201 accessions collected were analysed using the 20 nuclear SSR markers. A total of 120 alleles were scored from 20 loci. The number of alleles per locus varied from three (*mCrCIR06B05*) to ten (*mCrCIR03C08*) with an average of six (Table 1). The sample of 201 local accessions included four varietal groups (sour orange, sweet orange, lemon, and lime). Most of the markers, with the exception of *mCrCIR01D06a *and *mCrCIR02D09*, displayed very high heterozygosity values *Ho *(0.88 to 0.99).

**Table 1:** Genetic diversity of the Tunisian germplasm for 20 SSR markers

	Tunisian germplasm			Tunisian germplasm+ref
	
	n	Ho	He	N
*mCrCIR06B05*	3	0.891	0.494	3

*mCrCIR01D06a*	6	0.108	0.233	9

*mCrCIR02D04B*	4	0.881	0.554	9

*MEST458*	5	0.985	0.614	10

*MEST121*	4	0.911	0.549	5

*mCrCIR03D12a*	6	0.950	0.584	9

*mCrCIR02D09*	6	0.098	0.234	9

*MEST431*	5	0.990	0.586	8

*mCrCIR07D06*	6	0.965	0.615	8

*mCrCIR02G12*	5	0.965	0.585	7

*mCrCIR01F08a*	5	0.970	0.584	5

*mCrCIR01F04a*	6	0.980	0.618	11

*mCrCIR01C06*	8	0.980	0.649	9

*mCrCIR03G05*	7	0.975	0.626	9

*mCrCIR01C07*	5	0.970	0.613	8

*mCrCIR02A09*	9	0.901	0.609	8

*mCrCIR03C08*	10	0.995	0.615	12

*mCrCIR01H05*	7	0.921	0.560	8

*mCrCIR01E02*	7	0.911	0.546	7

*mCrCIR07D07*	6	0.970	0.608	8

All loci	120	0.866 +/-0.115	0.554 +/-0.050	162

n: allele number per locus for Tunisian germplasm; Ho: observed heterozygosity; He: expected heterozygosity; confidence interval at 95%; N: allele number per locus for Tunisian germplasm and reference accessions

This strong heterozygosity excess is coupled with low levels of inter-varietal polymorphism within groups. The lowest allelic diversity observed within the groups was found in sweet orange with 1.75 alleles per locus (Table 2). The three other groups displayed approximately two alleles per locus for lemon and three alleles per locus for lime and sour orange. The observed heterozygosity average is significantly higher than expected one on the whole sample (Table 1) and within sweet orange, sour orange and lemon (Table 2).

**Table 2:** Genetic diversity of the four varietal groups prospected in Tunisia

	na	G/N	He	Ho
Sour oranges	3.10	10/169	0.45 +/-0.07	0.88 +/-0.13

Oranges	1.75	1/12	0.37 +/-0.10	0.75 +/-0.19

Lemons	1.90	2/12	0.42 +/-0.08	0.85 +/-0.16

Limes	3.15	3/8	0.50 +/-0.09	0.63 +/-0.15

na: mean number of allele/locus; G: MLGs number; N: total number of prospected accessions; Ho: observed heterozygosity; He: expected heterozygosity;

Confidence interval at 95%

#### Genotype diversity

Among the 201 analysed accessions, only 16 distinct MLGs were obtained for the 20 loci studied yielding a *G/N *ratio of 0.079. Considering each varietal group (Table 2), the number of MLGs identified from 169 sour orange, 8 lime, 12 lemon and 12 sweet orange accessions were 10, 3, 2 and 1, respectively. It is notable that the majority (160) of the sour orange accessions corresponded to two MLGs, 'sour orange A' and 'sour orange B', representing 128 and 32 accessions, respectively. These two genotypes differed only in a single allele at locus *mCrCIR01C06*. Sour orange C, representing two accessions, differed from sour orange A only by its homozygosity at one locus (*mCrCIR02G12*). Interestingly, five of the other sour orange MLGs, each corresponding to one accession (sour orange D, E, F, J, H), displayed approximately half of the heterozygosity of the primary sour orange MLG without additional alleles. These five accessions may have arisen from zygotic seedlings following self-pollination. The two last sour orange MLGs (G and I) displayed intermediate proportions of heterozygous loci (78.6% and 65%, respectively) and a high number of 'new' alleles (11 and 12 alleles, respectively) not shared with the sour orange groups A, B and C.

The four 'Chiiri' limes displayed the same genotype for the 20 analysed loci. The two 'Sweet' limes shared the second lime MLG, the third lime MLG being represented by 'Arbi' and 'Beldi lemon'. Eleven of the 12 lemon accessions were identical, and the last one differed only at a single locus.

### Comparison of local germplasm with reference accessions

#### Nuclear markers

**Figure 1:**
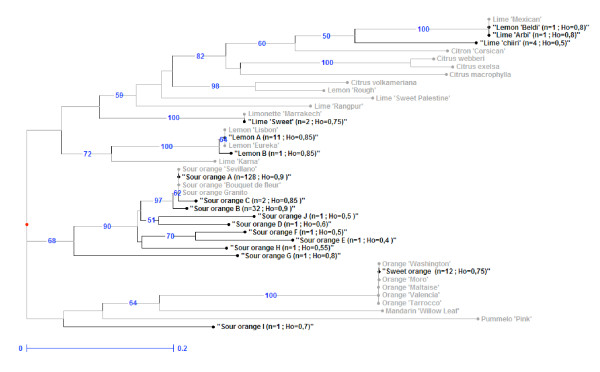
**Rooted Neighbour Joining tree illustrating relationships between Tunisian MLGs and reference accessions analysed with 20 nuclear SSR loci**. Bootstrap values (1000 replicates) are shown next to the branches if > 50. Tunisian MLGs names are coloured in black and reference accessions in grey. n: number of accessions sharing the MLG; Ho: observed heterozygosity. Reference accession names are indicated in Additional file 1b.

To assess the relationship of local accessions with reference genotypes of the same varietal groups, the 16 Tunisian MLGs and 23 citrus reference cultivars were analysed together using the same 20 SSR markers using capillary electrophoresis. The results were double checked to detect possible discrepancies with patterns observed with radio-labelled primers. They were found consistent both for the allele differentiated and the genotyping of all individuals. Accordingly, a NJ tree was established based on simple matching dissimilarity from allelic data (Figure 1). To analyse the potential origin of Tunisian genotypes showing variation from the reference MLGs, the data was scrutinized to determine (i) the number of loci differentiating these genotypes from the reference, (ii) the percentage of loci homozygous for one allele of the reference, (iii) the number of alleles not observed in the corresponding reference MLG, and (iv) the number of alleles shared with genotypes other than the considered reference (Table 3).

**Table 3:** Different loci and alleles distinguishing local accessions from the references

				Loci different from the corresponding reference MLG				
**Genotypes**	**N**	**Ho**	**Considered MLG reference**	**NL**	**HoD**	**NA**	Other reference MLG sharing these alleles*	**Potential origin**

Sour Orange A (id ref)	128	10%	Sevillano Sour Orange (IVIA-117)					
		
Sour Orange B	32	10%		1	0%	1	none	Mutation
		
Sour Orange C	2	15%		1	100%	0		Mutation
		
Sour Orange D	1	40%		6	100%	0		Selfing
		
Sour Orange E	1	60%		10	100%	0		Selfing
		
Sour Orange F	1	50%		8	100%	0		Selfing
		
Sour Orange J	1	50%		8	100%	0		Selfing
		
Sour Orange H	1	45%		7	100%	0		Selfing
		
Sour Orange G	1	20%		14	21%	11	mandarin (10), lime Rangpur (4), Orange (3), Rough lemon (4)...	hybridization with mandarin
		
Sour Orange I	1	30%		17	35%	12	Sweet orange (12), mandarin (5), pummelo (3)...	hybridization with orange

Lemon A (id ref)	11	15%	Eureka					

Lemon B	1	15%	lemon (IVIA-297)	1	0%	1	orange(1), sweet lime(1), mandarin (1)	Mutation

Lime Chiiri	4	50%	Mexican lime (IVIA-164)	14	50%	9	citron (8), lemon (6)	hybridization with citron

N: Number of accession; Ho: % of homozygous loci; NL: number of loci; HoD: % of homozygous loci among the one differentiated from the reference; NA: number of alleles not found in the reference MLG; * the number of shared alleles are indicated between parenthesis.

The clustering was generally consistent with varietal group classification. One cluster consisted of nine Tunisian sour orange MLGs and the three reference sour oranges. These three reference accessions and the most representative Tunisian MLG 'Sour orange A' were identical. The sour orange MLG G exhibited 14 loci which differed from the reference. Eleven alleles were not observed in the sour orange reference accessions. Ten of these alleles (91%) were found to be shared with 'Willow leaf' mandarin. Sour orange MLG I showed 12 alleles that were not observed in the sour orange references, although these 12 alleles are present in sweet oranges. These results suggest that G and I are hybrids of sour orange and mandarin, and of sour orange and sweet orange, respectively.

The sweet orange MLG from Tunisia and all sweet orange reference cultivars were identical. Furthermore, the MLG clustered with the 'Willow leaf' mandarin included as a *C. deliciosa *reference and with Pummelo 'Pink' included as the *C. maxima *reference.

All acid citrus (limes and lemons) clustered together. The 'lemon A' MLG, representing 11 of the 12 Tunisian lemon accessions, was identical to the two lemon references. The 'Lemon Beldi'/lime 'Arbi' MLG was identical to the 'Mexican' lime reference. Lime 'Chiiri' was closely related to these genotypes (bootstrap value of 50%) but differed from all the reference genotypes included in this study. Indeed, 'Chiiri' lime displayed 14 loci (50% of them were homozygous), which varied from the Mexican lime reference. This included nine alleles not observed in the Mexican lime, with eight (88.9%) of these being common to Corsican citron (Table 3). The 'sweet lime' MLG was identical to the 'limonette de Marrakech' genotype. When considering the results obtained from the Tunisian MLG and references (Table 1), the number of alleles for the 20 SSR locus increased from 120 for the Tunisian germplasm (six alleles per locus) to 162 (8.1 alleles per locus). All 120 alleles found in the Tunisian germplasm were also encountered in the reference set. However, 11 among the 16 local genotypes were not represented in the reference set.

#### Mitochondrial markerss

**Figure 2:**
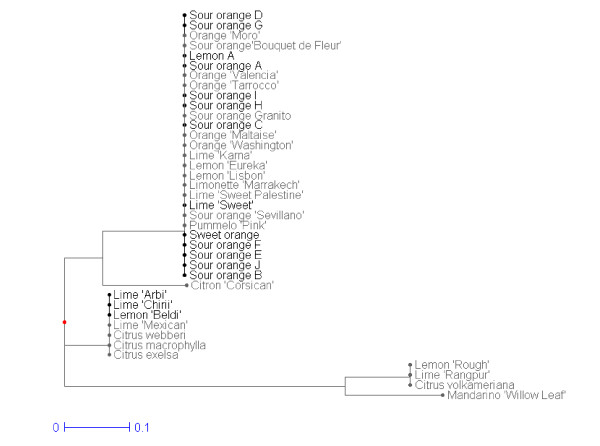
**Maternal relationships between Tunisian genotypes and reference accessions analysed with four indel mitochondrial markers (NJ tree)**. Tunisian MLGs names are coloured in black, reference accessions are in grey.

The 39 accessions analysed with nuclear SSR markers were also genotyped with rrn5/rrn18-, nad2/4-3, nad5/2-1 and nad7/1-2 mitochondrial markers. Five mitotypes were observed (Figure 2). The most representative included all sweet oranges, sour oranges and lemon, along with pummelo 'Pink', lime 'Karna', lime 'Sweet palestine', and lime 'Sweet' of Tunisia. A second mitotype associated lime 'Arbi'/lemon 'Beldi' and lime 'Chiiri', both from Tunisia, with 'Mexican' lime, *C. webberi, C. macrophylla *and *C. excelsa*. 'Rangpur' lime, 'Rough' lemon and 'Volkamer' lemon constituted a third cluster. The mandarin 'Willow leaf' and 'Corsican' citron represented the two last mitotypes.

## Discussion

### Inter-specific differentiation is the main structuring factor of Tunisian germplasm diversity

Population genetic parameters and cluster analysis indicated a high genetic structuring of the citrus Tunisian germplasm. This was also characterised by a significant excess of heterozygosity, both in the whole sample and in each varietal group (sour orange, sweet orange, lime and lemon). These results evidenced the important differentiation between the four varietal group analysed (sweet orange, sour orange, lime and lemons) and the limitation of gene flow between these groups.

The set of 20 SSR markers used in this study was sufficient to obtain genetic aggregations, enabling the clear identification of varietal groups of citrus and producing results consistent with previous studies [7,9,15,16]. The clustering of the Tunisian MLGs and reference cultivars confirmed the importance of inter-specific differentiation as a major element of the Tunisian germplasm variability. Indeed, the Tunisian accessions were split within three clusters. The first cluster included the majority of the Tunisian sour orange MLGs with the sour orange reference. All sweet orange accessions, including the Tunisian MLGs and reference, were identical and grouped in the second cluster, along with 'Willow Leaf' mandarin and 'Pink' pummelo. This grouping is in accordance with the origin of sweet orange, believed to have originated, as sour orange, from hybridizations between mandarin and pummelo gene pools [7]. When analysing mitochondrial markers, sweet orange and sour orange were found to be identical to the pummelo mitotype (Figure 2). This supports the previous hypothesis that pummelo acted as the female parent of sweet and sour oranges [7,8]. A third cluster grouped limes and lemons with citron and the other acid citrus species. All Tunisian lime and lemon were identical to the reference samples of the Spanish and French collection, with the exception of 'Chiiri' lime and the lemon MLG B. The currently accepted hypothesis is that lemon and lime are the result of secondary diversification by interspecific hybridization involving citron as one of the parents [7,15]. Nicolosi *et al. *[7] and Gulsen and Roose [30] proposed that lemon arose via hybridization between sour orange and citron. The mitotype analysis presented here confirms the hypothesis that sour orange was the maternal parent [7,8]. The numerous and complex origin of the lime cultivars will be discussed further with the source of intra-specific diversity in the Tunisian germplasm. All *citrus *species are sexually compatible [17,18]. However, vegetative horticultural propagation methods and facultative apomixis of the considered genotypes have undoubtedly prevented genetic mixing in Tunisia. Therefore, the multiple introductions (from different highly heterozygous secondary species) and the limitation of sexual recombination underlie a genetic diversity organization mainly based on inter-specific differentiation and very high heterozygosity.

### Intra-specific diversity and the source of variation in Tunisian germplasm

The observed heterozygosity within each varietal group is very high. This heterozygosity is associated with low inter-cultivar differentiation within species (low MLG/accession ratios). These results are consistent with the generally accepted inter-specific origin of these varietal groups [6,7,9,10,14,15] and the predominant role of mutation or epigenetic variation in their inter-specific differentiation [10,31].

#### Sour orange

Genotypic analysis of the 169 Tunisian sour orange accessions, at 20 microsatellite loci, revealed ten unique genotypes with important redundancies (G/N = 0.059). These redundancies were expected, as standard vegetatively-propagated rootstock varieties are distributed throughout the country. Thus, 162 accessions were found to correspond to three highly heterozygous, very closely clustered MLGs that differed only in a single allele. The most represented MLG (128 accessions) was identical to the three reference genotypes originating from other countries in the Mediterranean basin (Morocco, Spain, and Corsica). Within this group, different accessions were collected and named according to peculiar morphological traits. For example, the accessions 'sour orange Chiiri', 'sour orange with flattened fruits', 'sour orange with very small fruits and leaves', and sour oranges '*Arbi*' and '*Souri*' shared identical MLG A. It is suggested that the phenotypical variants observed between and within these three MLGs may be somatic mutants of the ancestral sour orange. The seven other sour orange MLGs corresponded to single accessions. All of them shared the same mitotype as the reference sour orange. Five of these MLGs exhibit reduced heterozygosity compared with the reference sour orange, as well as an absence of additional alleles when compared with the three predominant MLGs. It is highly probable that these MLGs are zygotic plants resulting from self-pollination within the predominant sour orange groups. The two remaining sour orange MLGs are likely sour orange × mandarin and sour orange × sweet orange hybrids. Six of the seven sour orange MLGs of zygotic origin were collected in Sbikha (Kairouan) from an orchard of mother trees used for seed propagation. All sour orange trees in this orchard were grown from seed. The other 22 plants analysed from this orchard genetically conformed to the predominant sour orange MLG. Thus, a rate of 21.4% zygotic plants can be estimated in this population. Facultative apomixis of citrus results from adventitious nucellar polyembryony in seeds also containing one zygotic embryo. The frequency of apomictic plants among seedlings is determined by the competition between nucellar embryos and the zygotic embryo during seed development and seed germination [19], as well as genotype and environment [19,32]. Frost and Soost [33] reported zygotic rates of 15% for sour orange, while Moore and Castle [34] did not find any zygotics in sour orange seedlings. Observations from the Sbikha seed park affirm the need to control the genetic conformity of seedling-produced mother trees in a propagation scheme. It also underscores the biological potential of diversification through sexual recombination, even in areas where only facultative apomictic genotypes are present. However, among all the included accessions, these zygotic plants have been observed as unique accessions and in a very specific context. These results imply that nursery workers and growers exert highly efficient counter-selection against these zygotic off-types by visual observation.

#### Sweet orange

The sweet orange accessions collected in Tunisia, although identified by phenotypic characters of the fruit (sweet, acid, red-coloured) or reported for their resistance to salinity (cv Meski), were identical for all analysed markers and highly heterozygous. Moreover, the sweet orange accessions also exhibited the same molecular profile as the five reference cultivars from the IVIA collection. This narrow genetic basis of the sweet orange accessions has been recognized in previous studies [13,31,35,36]. As for sour orange, most authors believe that sweet orange had interspecific origin (between pummelo and mandarin gene pools), and the inter-varietal diversity within this species is attributed to somatic mutations [10,15,31,37]. These mutations may alter horticultural characters, mostly fruit traits [38], and thus have been selected by man.

#### Lemons

Lemons were highly heterozygous for the SSR loci analysed. The Tunisian accessions displayed two MLGs. The main one (A) was identical to 'Eureka' and 'Lisbon' lemon reference accessions, and the second displayed only one different SSR allele, along with a marked mammiform apex of a pyriform fruit. The latter type most probably resulted from mutations of the reference lemons.

#### Limes

Three different MLGs were identified for lime in Tunisia. Although easily identified by the farmers due to their phenotypic differences, the Tunisian 'Arbi' and 'Beldi' limes exhibited the same genotype as the 'Mexican lime' reference accession, of which they may be phenotypic variants. These two Tunisian limes are therefore part of *C. aurantifolia *Swing. (Tanaka classification). The sweet lime accessions did not differ from 'limonette de Marrakech', a lime from Morocco and are classified in *C. limetta *Risso (Tanaka classification). The very close phenotypic relationship of the Tunisian sweet lime and 'limonette de Marrakech' was mentioned by Hodgson [38]. For this author, the only difference was the acidless flesh of the Tunisian lime while acidity was present in the 'limonette de Marrakech'. From our results, the Tunisian sweet lime is very likely an acidless mutant of an acid form such as 'limonette de Marrakech' as proposed by Hodgson. The 'Chiiri' lime MLG was not observed among the reference genotypes and clustered between the 'Mexican lime' genotype and the citron. It displayed a low heterozygosity level compared to the other citrus groups. A locus by locus comparison with 'Mexican lime' revealed a higher proportion of homozygous loci (50% versus 20%) and seven different alleles, six of which were shared with the reference citron. It is proposed that the Tunisian 'Chiiri' lime may have resulted from a cross between the 'Mexican' lime or one of its 'mutants' and citron. A recent study based on mitochondrial markers [8] suggested that limes have three different maternal origins: the *C. maxima *mitotype is shared with 'Marrakech limonette' and 'Palestine sweet lime', the *C. micrantha *mitotype is shared with 'Mexican' lime, and the acid mandarin (*C. reshni*) mitotype is shared with 'Rangpur' lime. In our analysis, the mitochondrial data confirmed the identities found at the nuclear level between limonette de Marrakech and Tunisian sweet limes as well as between Mexican lime and Beldi' lemon and 'Arbi' lime. 'Chiiri' lime also shared the same cytoplasm with 'Mexican' lime, which is consistent with Mexican lime being its maternal parent.

The analysis of Tunisian germplasm using microsatellites confirmed that the intra-specific diversification of the four apomictic species has resulted from three processes: (1) multiple introductions from diversified material, (2) mutation of local material, and (3) sexual recombination. The contributions of each of these processes varied depending on the species considered. For sweet oranges and lemons displaying very high heterozygosity and no or very little intra-group genetic diversity, sexual recombination can be discarded. For sour orange, sexual recombination was at the origin of seven accessions among 169 analysed (4%). For lime, the identities of two Tunisian MLGs with references from other countries points to a minimum of two introductions. The 'Chiiri' lime potentially arose from sexual events in Tunisia. However, it is difficult, without traceability of plant origin, to distinguish between multiple introductions of pre-differentiated materials or a *de novo *variation in Tunisia. These results show that, although rare, sexual reproduction occurs, and the progeny of self or inter-specific crossing may be selected by growers. However, it appears that mutation or epigenetic variation were the major factor of diversification within the different varietal groups, as previously underscored for other apomictic species [39,40]. The contribution of somatic mutations to the evolution of other vegetatively propagated crops, such as grapes [41], olives [42], yams [43] and cassava [44], has also been demonstrated. Human selection of new phenotypes and further clonal propagation are also key factors underlying the relatively high inter-varietal morphological polymorphism [6,10].

## Conclusions

In Tunisia, sour orange is found in nearly all citrus growing areas and is well adapted to environmental conditions. Lemon and lime represent other species traditionally used as self-rooted trees or as rootstocks, mainly in southern Tunisia, where rainfall is only 120 to 200 mm per year and most irrigation water is brackish. In this region, irrigation water can contain up to 4-7 g/l of salt, especially during the summer. These rootstocks are well-adapted to local conditions and bear full flavoured fruits. Farmers consider the 'Chiiri' lime variety to be the best adapted citrus rootstock to local bioclimatic conditions (salinity and drought) and report that it has better water uptake ability in comparison to sour orange. This study revealed that Tunisian citrus rootstock diversity has resulted from multiple introductions, mutations and residual sexual recombination of apomictic genotypes, as well as the impact of human selection. Original germplasm has been identified, particularly mutants and zygotic plants (mostly from selfing) of sour oranges, phenotypical variants of Mexican limes and a probable hybrid between a Mexican lime type and citron ('Chiiri' lime). This variability may be exploited either directly by nursery men and growers or by breeders to combine resistance to biotic and abiotic stresses with improvements in horticultural behaviour through conventional breeding or biotechnological approaches, such as somatic hybridization [2,45,46]. This study indicates the value of an accurate physiological and agronomic evaluation of the different MLGs identified with particular emphasis for tolerance to biotic and abiotic stresses and agronomic behaviour under unfavourable environmental conditions.

## Materials and methods

### Plant materials

**Figure 3:**
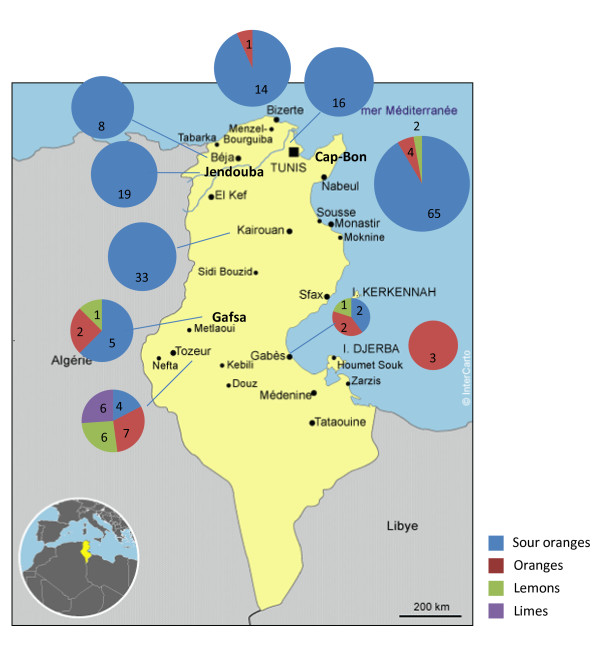
**Geographical distribution of the analysed Tunisian accessions**. Local Citrus accessions belonging to four species (*C. aurantium*, sour orange in blue; *C. sinensis*, orange in red; *C. limon*, lemon in green; and *C. aurantifolia*, lime in purple) were collected in main Tunisian regions. The number of each species accessions collected is represented in corresponding coloured sector within circle. Names of accessions for each sector correspond to the ones in Additional file 1a.

The rootstocks used in this study were collected from different regions of the country (Figure 3), including the main production area (Cap-Bon) and regions involved in production for local markets or domestic consumption (Southern, Northern Tunisia and oasis). Landraces were also sampled, as they are well adapted to local conditions. The sampling was performed according to geographical location and morphological diversity. Two hundred and one accessions were sampled for diversity analysis (Additional file 1a). The collected samples consisted of 169 sour oranges, 12 non-grafted sweet oranges, 12 non-grafted lemons and eight limes. Limes are well adapted to oasis conditions and represent the most popular rootstock of southern Tunisia (Tozeur, Gafsa, Gabes, Nefta). A local lime cultivar inappropriately called 'Beldi lemon' by growers was included in this analysis. To compare the Tunisian genotypes with known references, a set of 23 varieties (Additional file 1b) was selected. These reference varieties represent the different species analysed in Tunisia and the three main ancestral taxa of cultivated citrus (*C. maxima, C. medica *and *C. reticulata*) from the IVIA (Spain) and INRA/CIRAD (France) germplasm banks.

### Methods

#### Molecular markers

Twenty nuclear SSRs (Table 4) were selected based on their high polymorphism in previous studies of INRA/CIRAD and IVIA germplasm banks. The main criterion was the allele number. Seventeen SSRs were obtained from genomic libraries [24] and three from EST database mining [26].

**Table 4:** Primers of the 20 SSR loci used in the analysis

Locus name	EMBL **Accession No**.	Primer sequence Forward	Primer sequence Reverse	Repeat motifs	*Ta* (°*C*)
*mCrCIR02A09*	FR677568	ACAGAAGGTAGTATTTTAGGG	TTGTTTGGATGGGAAG	(GA)9	55

*mCrCIR03C08*	FR677576	CAGAGACAGCCAAGAGA	GCTTCTTACATTCCTCAAA	(GA)16	55

*mCrCIR02D04B*	FR677564	CTCTCTTTCCCCATTAGA	AGCAAACCCCACAAC	(GT)10(GA)7	50

*mCrCIR02D09*	FR677569	AATGATGAGGGTAAAGATG	ACCCATCACAAAACAGA	(GA)10	55

*mCrCIR03D12a*	FR677577	GCCATAAGCCCTTTCT	CCCACAACCATCACC	(GT)10(GA)6	50

*mCrCIR03G05*	FR677578	CCACACAGGCAGACA	CCTTGGAGGAGCTTTAC	(GAAA)3(GA)11	50

*mCrCIR02G12*	FR677575	AAACCGAAATACAAGAGTG	TCCACAAACAATACAACG	(GA)14	55

*MEST121*	DY275927	TCCCTATCATCGGCAACTTC	CAATAATGTTAGGCTGGATGGA	(TAA)9	55

*MEST431*	DY285140	GAGCTCAAAACAATAGCCGC	CATACCTCCCCGTCCATCTA	(CAG)7	55

*MEST458*	DY283417	CCCCCTCTTTTTCTCTTCCA	TTCTGGGCTGGTAGGTTCAG	(TC)12	55

*mCrCIR01C06*	AJ567393	GGACCACAACAAAGACAG	TGGAGACACAAAGAAGAA	(GA)9	50

*mCrCIR01C07*	AJ567394	GTCACTCACTCTCGCTCTTG	TTGCTAGCTGCTTTAACTTT	(CT)10	55

*mCrCIR01D06a*	AM489734	GATCAAAACATTATTCCAA	TTTTTCATCAACAAGACTG	(CA)12	50

*mCrCIR01E02*	AM489735	TGAATGGTACGGGAAATGC	CAGGGTCGGTGGAGAGGAT	(GA)16	55

*mCrCIR01F04a*	AM489736	AAGCATTTAGGGAGGGTCACT	TGCTGCTGCTGTTGTTGTTCT	(CT)13CC(CT)7	55

*mCrCIR01F08a*	AM489737	ATGAGCTAAAGAGAAGAGG	GGACTCAACACAACACAA	(GAAT)6	50

*mCrCIR01H05*	AJ567401	AAAACAACCAAAAGGACAAGATT	TTCAAACTAAACAAACCAACTCG	(GA)9	55

*mCrCIR06B05*	AM489744	GAACGATGGAATGAAGTG	ATGTTGATTACGAGACCTT	(GA)26	55

*mCrCIR07D06*	FR677581	CCTTTTCACAGTTTGCTAT	TCAATTCCTCTAGTGTGTGT	(TAAT)4N(TG)8(GA)11	55

*mCrCIR07D07*	AM489748	GCTGATGATACGCACGAACC	CACAACGCCAAAAACGACTC	(GA)10	55

*Ta: *annealing temperature. MEST: Marker selected from Clementine Expressed Sequence Tag, mCrCIR: Microsatellite selected from genomic sequences of *C. reticulata at CIRAD*

Four mitochondrial indel markers (rrn5/rrn18-1, nad2/4-3, nad5/2-1, and nad7/1-2), developed by Froelicher *et al. *[8], were also used to analyse the maternal phylogeny of the Tunisian germplasm. These markers allowed a clear differentiation among the four basic taxa of cultivated citrus. Moreover they differentiated 3 mitotypes for limes and were therefore well adapted to the objectives of the study.

#### Molecular marker analysis

Initially, the 201 collected accessions were genotyped using 20 polymorphic SSR loci at CIRAD (Montpellier, France). Primers were radio-labelled with ^33^P prior to PCR amplification. The PCR reaction for SSR analysis was performed in a total volume of 20 μl containing 25 ng genomic DNA, 0.2 μM of each primer, 200 μM of each dNTP, 2 μl of 10X PCR buffer (100 mM Tris-HCl pH 8.3, 500 mM KCl, 15 mM MgCl_2_, 0.01% glycerol), and 0.75 units of Taq DNA polymerase. PCR reactions were performed using a MJ research thermal cycler model PTC-100™ and the following parameters: an initial denaturation at 94°C for 5 min, 35 amplification cycles of 30 s at 94°C, 1 min at 50-55°C (depending on the primer pair), 30 s at 72°C, and a final elongation at 72°C for 4 min. PCR products were visualized by running 6 μl of each sample on 5% polyacrylamide denaturing gels. Gels were dried and exposed to autoradiographic film. To minimize genotyping errors and for further comparison, all gels included a control individual (sour orange 'Granito') in four gel lanes and a ladder as a size standard. Unclear genotypes were systematically reanalysed.

Using the same 20 nuclear SSR markers and four Indels mitochondrial markers, the different multi-locus genotypes (MLGs) observed in the initial analysis were reanalysed along with the reference set of 23 accessions. These analyses were conducted using capillary electrophoresis (CEQ™ 8000 Genetic Analysis System) at IVIA (Valence, Spain) and repeated two times. The PCR reaction was performed using 2 ng/μl of template DNA, 0.2 mM of dNTPs, 0.2 μM of WellRED dye-labeled forward primer, 0.2 μM of reverse primer, 10X PCR buffer (Fermentas), 1.5 mM of MgCl_2 _and 0.5 U/μl of Taq DNA polymerase (Fermentas). The PCR conditions were as follows: 5 min at 94°C; 40 cycles of 30 s at 94°C, 30 s at 55°C (or 50°C depending on the primer) and 1 min at 72°C; and 4 min at 72°C.

#### Analysis of genetic data

For each primer, bands (PAGE) or peaks (capillary electrophoresis) were scored as allelic data. The allelic pattern of each accession was cross-checked using two readers.

These data were used to calculate a genetic dissimilarity matrix using the simple matching dissimilarity index (d_ij_) between pairs of accessions (units) [47].

$${\text{d}}_{\text{ij}}=1-\frac{1}{L}\sum_{l=1}^{L}\frac{m_{l}}{2}$$

where d_ij _represents the dissimilarity between units i and j, L represents the number of loci, and m_l _represents the number of matching alleles between i and j for locus l. From the dissimilarity matrix, a Neighbor-Joining tree [48] was computed using the DARwin software version 5.0.158 (Dissimilarity Analysis and Representation for Windows, http://darwin.cirad.fr/darwin[49]. Branch robustness was tested using 1000 bootstraps.

GENETIX software 4.05.2 http://www.genetix.univ-montp2.fr/genetix/genetix.htm[50] was used to estimate several parameters describing the genetic diversity and its organization within and between the four varietal groups encountered in Tunisia (sour orange, sweet orange, lemon and lime). The parameters estimated for each marker were the allele number (n), the mean number of alleles, allele frequencies, observed and expected mean heterozygosities (*Ho *and *H*e, respectively). The number of distinct MLGs in each varietal group was identified from the whole set of markers.

## Competing interests

The authors declare that they have no competing interests.

## Authors' contributions

HS collected the germplasm for this study, carried out the molecular characterization using radio-labelled markers at CIRAD, participated in the data analysis and drafted the manuscript. MFD participated in the design of the study, coordinated the data analysis and helped draft the manuscript. AGL carried out capillary electrophoresis analyses at IVIA. ZB participated in the design of the study and coordinated the collecting part. YF provided the primers used in this study and participated in the data analysis. AMR supervised the molecular characterization at CIRAD. XP and JPJC supervised the statistical analyses. LN furnished the reference germplasm accessions from IVIA collection and participated to the manuscript. MH contributed to the collecting part of the study. PO conceived the study, coordinated the project and contributed to draft the manuscript. All authors read and approved the final manuscript.

## Supplementary Material

Additional_files 1 **Additional file **1a**Tunisian local germplasm sampled for diversity analysis (201 accessions) from different varietal group**. Additional file 1b Reference varieties (23 accessions) from different taxa. Click here for file
