# Supplementary material for: Assessment of the genetic diversity of the Tunisian citrus rootstock germplasm
Source: BMC Genet. 2012 Mar 19;13:16. doi: 10.1186/1471-2156-13-16 (PMC3323426; doi:10.1186/1471-2156-13-16)
Supplement: Additional_files 1 — Additional file 1aTunisian local germplasm sampled for diversity analysis (201 accessions) from different varietal group. Additional file 1b Reference varieties (23 accessions) from different taxa. [file 1471-2156-13-16-S1.PDF]

Additional files 1

Additional file 1a - Tunisian local germplasm sampled for diversity analysis (201 accessions) from different varietal group

Accession number	Local accession name	Varietal group	Latin name	Tree origin	Origin
1	Sour orange SP1	Sour orange	*Citrus aurantium* L.	Seedling	Sbikha seed park- Kairouan
2	Sour orange SP2	Sour orange	*Citrus aurantium* L.	Seedling	Sbikha seed park- Kairouan
3	Sour orange SP3	Sour orange	*Citrus aurantium* L.	Seedling	Sbikha seed park- Kairouan
4	Sour orange SP4	Sour orange	*Citrus aurantium* L.	Seedling	Sbikha seed park- Kairouan
5	Sour orange SP5	Sour orange	*Citrus aurantium* L.	Seedling	Sbikha seed park- Kairouan
6	Sour orange SP6	Sour orange	*Citrus aurantium* L.	Seedling	Sbikha seed park- Kairouan
7	Sour orange SP7	Sour orange	*Citrus aurantium* L.	Seedling	Sbikha seed park- Kairouan
8	Sour orange SP8	Sour orange	*Citrus aurantium* L.	Seedling	Sbikha seed park- Kairouan
9	Sour orange SP9	Sour orange	*Citrus aurantium* L.	Seedling	Sbikha seed park- Kairouan
10	Sour orange SP10	Sour orange	*Citrus aurantium* L.	Seedling	Sbikha seed park- Kairouan
11	Sour orange SP11	Sour orange	*Citrus aurantium* L.	Seedling	Sbikha seed park- Kairouan
12	Sour orange SP12	Sour orange	*Citrus aurantium* L.	Seedling	Sbikha seed park- Kairouan
13	Sour orange SP13	Sour orange	*Citrus aurantium* L.	Seedling	Sbikha seed park- Kairouan
14	Sour orange SP14	Sour orange	*Citrus aurantium* L.	Seedling	Sbikha seed park- Kairouan
15	Sour orange SP15	Sour orange	*Citrus aurantium* L.	Seedling	Sbikha seed park- Kairouan
16	Sour orange SP16	Sour orange	*Citrus aurantium* L.	Seedling	Sbikha seed park- Kairouan
17	Sour orange SP17	Sour orange	*Citrus aurantium* L.	Seedling	Sbikha seed park- Kairouan
18	Sour orange SP18	Sour orange	*Citrus aurantium* L.	Seedling	Sbikha seed park- Kairouan
19	Sour orange SP19	Sour orange	*Citrus aurantium* L.	Seedling	Sbikha seed park- Kairouan
20	Sour orange SP20	Sour orange	*Citrus aurantium* L.	Seedling	Sbikha seed park- Kairouan
21	Sour orange SP21	Sour orange	*Citrus aurantium* L.	Seedling	Sbikha seed park- Kairouan
22	Sour orange SP22	Sour orange	*Citrus aurantium* L.	Seedling	Sbikha seed park- Kairouan
23	Sour orange SP23	Sour orange	*Citrus aurantium* L.	Seedling	Sbikha seed park- Kairouan
24	Sour orange SP24	Sour orange	*Citrus aurantium* L.	Seedling	Sbikha seed park- Kairouan
25	Sour orange SP25	Sour orange	*Citrus aurantium* L.	Seedling	Sbikha seed park- Kairouan
26	Sour orange SP26	Sour orange	*Citrus aurantium* L.	Seedling	Sbikha seed park- Kairouan
27	Sour orange SP27	Sour orange	*Citrus aurantium* L.	Seedling	Sbikha seed park- Kairouan
28	Sour orange E1	Sour orange	*Citrus aurantium* L.	Seedling	Echriihaat- Cap-Bon
29	Sour orange E2	Sour orange	*Citrus aurantium* L.	Seedling	Echriihaat- Cap-Bon
30	Sour orange E3	Sour orange	*Citrus aurantium* L.	Seedling	Echriihaat- Cap-Bon
31	Sour orange E4	Sour orange	*Citrus aurantium* L.	Seedling	Echriihaat- Cap-Bon
32	Sour orange BK1	Sour orange	*Citrus aurantium* L.	Seedling	Beni- Khiar- Cap-Bon
33	Sour orange BK2	Sour orange	*Citrus aurantium* L.	Seedling	Beni- Khiar- Cap-Bon
34	Sour orange G1	Sour orange	*Citrus aurantium* L.	Seedling	El Gobba- Cap-Bon
35	Sour orange G2	Sour orange	*Citrus aurantium* L.	Seedling	El Gobba- Cap-Bon
36	Sour orange G3	Sour orange	*Citrus aurantium* L.	Seedling	El Gobba- Cap-Bon
37	Sour orange G4	Sour orange	*Citrus aurantium* L.	Seedling	El Gobba- Cap-Bon
38	Sour orange G5	Sour orange	*Citrus aurantium* L.	Seedling	El Gobba- Cap-Bon
39	Sour orange G6	Sour orange	*Citrus aurantium* L.	Seedling	El Gobba- Cap-Bon
40	Sour orange mother-tree MBZ1	Sour orange	*Citrus aurantium* L.	Seedling	Menzel bou Zelfa- Cap-Bon
41	Orange Meski "kalb" MBZ2	Sweet Orange	*Citrus sinensis* (L.) Osbeck	Seedling	Menzel bou Zelfa- Cap-Bon
42	Sour orange mother-tree MBZ3	Sour orange	*Citrus aurantium* L.	Seedling	Menzel bou Zelfa- Cap-Bon

43	Orange Meski “kalb” MBZ4	Sweet Orange	*Citrus sinensis* (L.) Osbeck	Seedling	Menzel bou Zelfa- Cap-Bon
44	Sour orange MBZ5	Sour orange	*Citrus aurantium* L.	Seedling	Menzel bou Zelfa- Cap-Bon
45	Sour orange with filiform leaves MCB1	Sour orange	*Citrus aurantium* L.	Seedling	Menzel bou Zelfa- Cap-Bon
46	Selected Sour orange MCB2	Sour orange	*Citrus aurantium* L.	Seedling	Menzel bou Zelfa- Cap-Bon
47	Sour orange A1	Sour orange	*Citrus aurantium* L.	Grafting	El Alia- Bizerte
48	Sour orange AB1	Sour orange	*Citrus aurantium* L.	Seedling	El Alia- Bizerte
49	Sour orange shoot AB2	Sour orange	*Citrus aurantium* L.	Seedling	El Alia- Bizerte
50	Sour orange AB3	Sour orange	*Citrus aurantium* L.	Seedling	El Alia- Bizerte
51	Sour orange GCB1	Sour orange	*Citrus aurantium* L.	Seedling	El Gobba- Cap-Bon
52	Sour orange GOC1	Sour orange	*Citrus aurantium* L.	Seedling	El Gobba- Cap-Bon
53	Sour orange GOC2	Sour orange	*Citrus aurantium* L.	Seedling	El Gobba- Cap-Bon
54	Sour orange AZ1	Sour orange	*Citrus aurantium* L.	Unknown	El Azib- Bizerte
55	Sour orange AZ2	Sour orange	*Citrus aurantium* L.	Unknown	El Azib- Bizerte
56	Sour orange ABZ1	Sour orange	*Citrus aurantium* L.	Unknown	El Azib- Bizerte
57	Sour orange ABZ2	Sour orange	*Citrus aurantium* L.	Unknown	El Azib- Bizerte
58	Sour orange BNk1	Sour orange	*Citrus aurantium* L.	Seedling	Beni- Khiar- Cap-Bon
59	Sour orange shoot BNk2	Sour orange	*Citrus aurantium* L.	Seedling	Beni- Khiar- Cap-Bon
60	Ordinary orange flower tree BNk3	Sour orange	*Citrus aurantium* L.	Seedling	Beni- Khiar- Cap-Bon
61	Sour orange BKA1	Sour orange	*Citrus aurantium* L.	Seedling	Beni- Khiar- Cap-Bon
62	Sour orange YTC1	Sour orange	*Citrus aurantium* L.	Seedling	El Gobba- Cap-Bon
63	Sour orange YTC2	Sour orange	*Citrus aurantium* L.	Seedling	El Gobba- Cap-Bon
64	Sour orange YTC3	Sour orange	*Citrus aurantium* L.	Seedling	El Gobba- Cap-Bon
65	Sour orange SMS1	Sour orange	*Citrus aurantium* L.	Seedling	Bou Argoub- Cap-Bon
66	Sour orange SMS2	Sour orange	*Citrus aurantium* L.	Seedling	Bou Argoub- Cap-Bon
67	Sour orange SMS3	Sour orange	*Citrus aurantium* L.	Seedling	Bou Argoub- Cap-Bon
68	Sour orange TS1	Sour orange	*Citrus aurantium* L.	Seedling	Bou Argoub- Cap-Bon
69	Sour orange TS2	Sour orange	*Citrus aurantium* L.	Seedling	Bou Argoub- Cap-Bon
70	Sour orange TS3	Sour orange	*Citrus aurantium* L.	Seedling	Bou Argoub- Cap-Bon
71	Sour orange 120-150 years old BA1	Sour orange	*Citrus aurantium* L.	Unknown	Bou Argoub- Cap-Bon
72	Sour orange 120-150 years old BA2	Sour orange	*Citrus aurantium* L.	Unknown	Bou Argoub- Cap-Bon
73	Sour orange 120-150 years old BA3	Sour orange	*Citrus aurantium* L.	Unknown	Bou Argoub- Cap-Bon
74	Sour orange RDJ1	Sour orange	*Citrus aurantium* L.	Seedling	Ras Djebel- Bizerte
75	Sour orange RDJ2	Sour orange	*Citrus aurantium* L.	Seedling	Ras Djebel- Bizerte
76	«Sweet» Sour orange RDJ3	Sour orange	*Citrus aurantium* L.	Seedling	Ras Djebel- Bizerte
77	Sour orange KB1	Sour orange	*Citrus aurantium* L.	Seedling	Kaa el Ballout- Bizerte
78	Sour orange KB2	Sour orange	*Citrus aurantium* L.	Seedling	Kaa el Ballout- Bizerte
79	Orange RB1	Sweet Orange	*Citrus sinensis* (L.) Osbeck	Seedling	Raf-Raf- Bizerte
80	"Edible" Sour orange "Chiiri" RB2	Sour orange	*Citrus aurantium* L.	Seedling	Raf-Raf- Bizerte
81	Sour orange BSJ1	Sour orange	*Citrus aurantium* L.	Seedling	Bou Salem- Jendouba
82	Sour orange FS1	Sour orange	*Citrus aurantium* L.	Seedling	Bou Salem- Jendouba
83	Sour orange INRA1	Sour orange	*Citrus aurantium* L.	Grafting	Bou Salem- Jendouba
84	Sour orange ABM1	Sour orange	*Citrus aurantium* L.	Seedling	Mornag
85	Sour orange ABM2	Sour orange	*Citrus aurantium* L.	Seedling	Mornag
86	Sour orange GIF1	Sour orange	*Citrus aurantium* L.	Seedling	Oued Mliz- Jendouba
87	Sour orange GIF2	Sour orange	*Citrus aurantium* L.	Seedling	Oued Mliz- Jendouba
88	Sour orange GIFJ1	Sour orange	*Citrus aurantium* L.	Seedling	Oued Mliz- Jendouba
89	Sour orange GIFJ2	Sour orange	*Citrus aurantium* L.	Seedling	Oued Mliz- Jendouba
90	Sour orange OTD1	Sour orange	*Citrus aurantium* L.	Seedling	El Kodia- Jendouba

91	Sour orange OTD2	Sour orange	*Citrus aurantium* L.	Seedling	El Kodia- Jendouba
92	Sour orange HK1	Sour orange	*Citrus aurantium* L.	Unknown	Haffouz- Kairouan
93	Sour orange HK2	Sour orange	*Citrus aurantium* L.	Unknown	Haffouz- Kairouan
94	Sour orange JK1	Sour orange	*Citrus aurantium* L.	Seedling	Ain Jalloula- Kairouan
95	Sour orange KDS1	Sour orange	*Citrus aurantium* L.	Grafting	Sbikha- Kairouan
96	Sour orange KDS2	Sour orange	*Citrus aurantium* L.	Grafting	Sbikha- Kairouan
97	Sour orange HJB1	Sour orange	*Citrus aurantium* L.	Grafting	Béjà
98	Sour orange LTB1	Sour orange	*Citrus aurantium* L.	Seedling	Thibar- Béjà
99	Sour orange with “small fruits” LTB2	Sour orange	*Citrus aurantium* L.	Seedling	Thibar- Béjà
100	Sour orange JPT1	Sour orange	*Citrus aurantium* L.	Unknown	Testour- Béjà
101	Sour orange JPT2	Sour orange	*Citrus aurantium* L.	Unknown	Testour- Béjà
102	Sour orange BCV1	Sour orange	*Citrus aurantium* L.	Unknown	Testour- Béjà
103	Sour orange VGB1	Sour orange	*Citrus aurantium* L.	Seedling	Testour- Béjà
104	Sour orange VGB2	Sour orange	*Citrus aurantium* L.	Seedling	Testour- Béjà
105	Sour orange ABMO1	Sour orange	*Citrus aurantium* L.	Seedling	Mornag
106	Sour orange ABMO2	Sour orange	*Citrus aurantium* L.	Seedling	Mornag
107	Sour orange ERR1	Sour orange	*Citrus aurantium* L.	Grafting	Mornag
108	Sour orange GIAF1	Sour orange	*Citrus aurantium* L.	Seedling	Mornag
109	Sour orange GIAF2	Sour orange	*Citrus aurantium* L.	Seedling	Mornag
110	Local “arbi” lemon grafted with “Maltaise” orange variety OTDCB1	Lemon	*Citrus limon* (L.) Burm.	Seedling	Menzel bou Zelfa- Cap-Bon
111	Sour orange OTDCB2	Sour orange	*Citrus aurantium* L.	Seedling	Menzel bou Zelfa- Cap-Bon
112	Sour orange OTDCB3	Sour orange	*Citrus aurantium* L.	Seedling	Menzel bou Zelfa- Cap-Bon
113	Sour orange OTDCB4	Sour orange	*Citrus aurantium* L.	Seedling	Menzel bou Zelfa- Cap-Bon
114	Sour orange with flattened fruits OTDCB5	Sour orange	*Citrus aurantium* L.	Seedling	Menzel bou Zelfa- Cap-Bon
115	Orange Meski “kalb” LMM1	Sweet Orange	*Citrus sinensis* (L.) Osbeck	Seedling	Menzel bou Zelfa- Cap-Bon
116	Orange Meski “kalb” LMM2	Sweet Orange	*Citrus sinensis* (L.) Osbeck	Seedling	Menzel bou Zelfa- Cap-Bon
117	Local “arbi” lemon grafted with “Maltaise” orange variety LMM3	Lemon	*Citrus limon* (L.) Burm.	Seedling	Menzel bou Zelfa- Cap-Bon
118	Sour orange CSK1	Sour orange	*Citrus aurantium* L.	Seedling	Sbikha- Kairouan
119	Sour orange HMA1	Sour orange	*Citrus aurantium* L.	Seedling	Mides- Tozeur
120	Orange “chroubou” HMA2	Sweet Orange	*Citrus sinensis* (L.) Osbeck	Seedling	Mides- Tozeur
121	Lime “Chiiri” HMA3	Lime	*Citrus aurantifolia* (Christm.) Swing.	Layering	Mides- Tozeur
122	Lime “Chiiri” HMA4	Lime	*Citrus aurantifolia* (Christm.) Swing.	Layering	Mides- Tozeur
123	Ordinary lemon MBA1	Lemon	*Citrus limon* (L.) Burm.	Layering	Mides- Tozeur
124	Sour orange MBA2	Sour orange	*Citrus aurantium* L.	Seedling	Mides- Tozeur
125	Bloody orange TT1	Sweet Orange	*Citrus sinensis* (L.) Osbeck	Seedling	Tamaghza- Tozeur
126	Orange Meski TT2	Sweet Orange	*Citrus sinensis* (L.) Osbeck	Seedling	Tamaghza- Tozeur
127	Lime “Chiiri” CHD1	Lime	*Citrus aurantifolia* (Christm.) Swing.	Layering	Degache- Tozeur
128	Lemon CHD2	Lemon	*Citrus limon* (L.) Burm.	Layering	Degache- Tozeur
129	Lemon “Beldi” CHD3	Lime	*Citrus aurantifolia* (Christm.) Swing.	layering	Degache- Tozeur
130	Orange CHD4	Sweet Orange	*Citrus sinensis* (L.) Osbeck	Seedling	Degache- Tozeur

131	Lemon with mammiform apex and pyriform fruits CHD5	Lemon	*Citrus limon* (L.) Burm.	Layering	Degache- Tozeur
132	Lemon MSD1	Lemon	*Citrus limon* (L.) Burm.	Layering	Degache- Tozeur
133	Sour orange SFG1	Sour orange	*Citrus aurantium* L.	Seedling	Gafsa
134	Sour orange mother tree SFG2	Sour orange	*Citrus aurantium* L.	Seedling	Gafsa
135	Sour orange SFG3	Sour orange	*Citrus aurantium* L.	Seedling	Gafsa
136	Lemon mother tree (Gafsa lime) SFG4	Lemon	*Citrus limon* (L.) Burm.	Layering	Gafsa
137	Sweet lime SFG5	Lime	*Citrus limetta* Risso	Layering	Gafsa
138	Sour orange HZG1	Sour orange	*Citrus aurantium* L.	Grafting	El Gsar- Gafsa
139	Sour orange HZG2	Sour orange	*Citrus aurantium* L.	Grafting	El Gsar- Gafsa
140	Lemon "Chiiri" HZG3	Lemon	*Citrus limon* (L.) Burm.	Layering	El Gsar- Gafsa
141	Sour orange JSK1	Sour orange	*Citrus aurantium* L.	Seedling	El Khlidia- Mornag
142	Sour orange JSK2	Sour orange	*Citrus aurantium* L.	Seedling	El Khlidia- Mornag
143	Sour orange JSK3	Sour orange	*Citrus aurantium* L.	Seedling	El Khlidia- Mornag
144	Sour orange SHM1	Sour orange	*Citrus aurantium* L.	Seedlingt	El Khlidia- Mornag
145	Sour orange "Arbi" HTH1	Sour orange	*Citrus aurantium* L.	Seedling	Hammamet
146	Sour orange " Souri" HTH2	Sour orange	*Citrus aurantium* L.	Seedling	Hammamet
147	Sour orange "Arbi" HTH3	Sour orange	*Citrus aurantium* L.	Seedling	Hammamet
148	Sour orange "Arbi" AKH1	Sour orange	*Citrus aurantium* L.	Seedling	Hammamet
149	Sour orange " Souri" AKH2	Sour orange	*Citrus aurantium* L.	Seedling	Hammamet
150	Sour orange "Arbi" grafted HMH1	Sour orange	*Citrus aurantium* L.	Seedling	El Ghrouss- Hammamet
151	Sour orange "Arbi" HMH2	Sour orange	*Citrus aurantium* L.	Seedling	El Ghrouss- Hammamet
152	Sour orange "Arbi" non-irrigated TJH1	Sour orange	*Citrus aurantium* L.	Unknown	Hammamet
153	Sour orange RFH1	Sour orange	*Citrus aurantium* L.	Grafting	Hammamet
154	Sour orange EXFD1	Sour orange	*Citrus aurantium* L.	Grafting	Hammamet
155	Sour orange EXFD2	Sour orange	*Citrus aurantium* L.	Grafting	Hammamet
156	Sour orange CHO1	Sour orange	*Citrus aurantium* L.	Seedling	Ouechtata- Béjà
157	Sour orange CHO2	Sour orange	*Citrus aurantium* L.	Seedling	Ouechtata- Béjà
158	Sour orange KOUB1	Sour orange	*Citrus aurantium* L.	Seedling	Ouechtata- Béjà
159	Sour orange KOUB2	Sour orange	*Citrus aurantium* L.	Seedling	Ouechtata- Béjà
160	Sour orange KOUB3	Sour orange	*Citrus aurantium* L.	Seedling	Ouechtata- Béjà
161	Sour orange KOUB4	Sour orange	*Citrus aurantium* L.	Seedling	Ouechtata- Béjà
162	Sour orange grafted KOUB5	Sour orange	*Citrus aurantium* L.	Seedling	Ouechtata- Béjà
163	Sour orange KOUB6	Sour orange	*Citrus aurantium* L.	Seedling	Ouechtata- Béjà
164	Sour orange KOUB7	Sour orange	*Citrus aurantium* L.	Seedling	Ouechtata- Béjà
165	Sour orange BOU1	Sour orange	*Citrus aurantium* L.	Seedling	Ouechtata- Béjà
166	Sour orange CCSPS1	Sour orange	*Citrus aurantium* L.	Seedling	Menzel bou Zelfa- Cap-Bon
167	Sour orange CCSPS2	Sour orange	*Citrus aurantium* L.	Seedling	Menzel bou Zelfa- Cap-Bon
168	Sour orange CCSPS3	Sour orange	*Citrus aurantium* L.	Seedling	Menzel bou Zelfa- Cap-Bon
169	Sour orange MJK1	Sour orange	*Citrus aurantium* L.	Grafting	El Khlidia- Mornag
170	Sour orange HBK1	Sour orange	*Citrus aurantium* L.	Seedling	El Khlidia- Mornag
171	Sour orange HBRK1	Sour orange	*Citrus aurantium* L.	Seedling	El Khlidia- Mornag
172	Sour orange FGK1	Sour orange	*Citrus aurantium* L.	Seedling	El Khlidia- Mornag
173	Sour orange SPAM1	Sour orange	*Citrus aurantium* L.	Seedling	El M'hamdia
174	Lemon BZT1	Lemon	*Citrus limon* (L.) Burm.	Layering	Old oasis- Tozeur
175	Lime "Arbi" MMT1	Lime	*Citrus aurantifolia* (Christm.) Swing.	Layering	Old oasis- Tozeur
176	Sweet lime ZAD1	Lime	*Citrus limetta* Risso	Layering	Degache- Tozeur
177	Orange Meski ZAD2	Sweet Orange	*Citrus sinensis* (L.) Osbeck	Seedling	Degache- Tozeur
178	Sour orange ZAD3	Sour orange	*Citrus aurantium* L.	Grafting	Degache- Tozeur

179	Orange “Chammeme karess” ABD1	Sweet Orange	*Citrus sinensis* (L.) Osbeck	Unknown	Degache- Tozeur
180	Orange “Chammeme hlou” ABD2	Sweet Orange	*Citrus sinensis* (L.) Osbeck	Unknown	Degache- Tozeur
181	Sour orange ABD3	Sour orange	*Citrus aurantium* L.	Grafting	Degache- Tozeur
182	Lime “Chiiri” ISSA1	Lime	*Citrus aurantifolia* (Christm.) Swing.	Layering	Degache- Tozeur
183	Lemon of the region ARRM1	Lemon	*Citrus limon* (L.) Burm.	Layering	Merith- Gabes
184	Sour orange ARROU1	Sour orange	*Citrus aurantium* L.	Grafting	Kettana- Gabes
185	Lemon ARROU2	Lemon	*Citrus limon* (L.) Burm.	Layering	Kettana- Gabes
186	Sour orange BMJ1	Sour orange	*Citrus aurantium* L.	Grafting	El-Mahboubine- Djerba
187	Sour orange ABDH1	Sour orange	*Citrus aurantium* L.	Grafting	El-Mahboubine- Djerba
188	Sour orange FFJ1	Sour orange	*Citrus aurantium* L.	Grafting	El-Mahboubine- Djerba
189	Sour orange MBCH1	Sour orange	*Citrus aurantium* L.	Grafting	Chnini- Gabes
190	Lemon MBCH2	Lemon	*Citrus limon* (L.) Burm.	Layering	Chnini- Gabes
191	Sour orange “Arbi” KJH1	Sour orange	*Citrus aurantium* L.	Seedling	Hammamet
192	Sour orange “Souri” KJH2	Sour orange	*Citrus aurantium* L.	Seedling	Hammamet
193	Sour orange “Arbi” SHJ1	Sour orange	*Citrus aurantium* L.	Seedling	Hammamet
194	Sour orange “Souri” SHJ2	Sour orange	*Citrus aurantium* L.	Seedling	Hammamet
195	Sour orange “Souri” OTHK1	Sour orange	*Citrus aurantium* L.	Seedling	Hammamet
196	Sour orange shoot (more than 50 years old) OTHK2	Sour orange	*Citrus aurantium* L.	Grafting	Hammamet
197	Sour orange SKAH1	Sour orange	*Citrus aurantium* L.	Seedling	Hammamet
198	Sour orange AMH1	Sour orange	*Citrus aurantium* L.	Seedling	Hammamet
199	Sour orange “Arbi” FMH1	Sour orange	*Citrus aurantium* L.	Seedling	Hammamet
200	Sour orange “Arbi” FGH1	Sour orange	*Citrus aurantium* L.	Seedling	Hammamet
201	Sour orange shoot FGH2	Sour orange	*Citrus aurantium* L.	Grafting	Hammamet

Additional file 1b - Reference varieties (23 accessions) from different taxa

Genotype	Latin name	Accession number
Granito Sour orange	*Citrus aurantium* L.	ICVN 0110015
‘Bouquet de fleurs’ Sour orange	*Citrus aurantium* L.	IVIA – 139
‘Sevillano’ Sour orange	*Citrus aurantium* L.	IVIA – 117
'Willow Leaf' Mandarin	*Citrus deliciosa* Ten.	IVIA – 154
‘Tarrocco’ Orange	*Citrus sinensis* (L.) Osbeck	IVIA – 271
‘Maltaise’ Orange	*Citrus sinensis* (L.) Osbeck	IVIA – 393
‘Moro’ Orange	*Citrus sinensis* (L.) Osbeck	IVIA – 44
‘Washington Navel’ Orange	*Citrus sinensis* (L.) Osbeck	IVIA – 222
‘Valencia Late’ Orange	*Citrus sinensis* (L.) Osbeck	IVIA – 126
C. excelsa	*Citrus excelsa Wester*	IVIA – 167
‘Mexican’ lime	*Citrus aurantifolia* (Christm.) Swing.	IVIA – 164
‘Marrakech’ Limonette	*Citrus limetta* Risso	IVIA – 484
‘Palestine sweet’ lime	*Citrus limettoïdes* Tan.	IVIA – 305
‘Rangpur’ Lime	*Citrus limonia* Osbeck	IVIA – 334
Volkamer lemon	*Citrus limonia* Osbeck	IVIA – 432
Alemow	*Citrus macrophylla* Wester	IVIA – 288
Citrus webberii	*Citrus webberii* Wester	IVIA – 234
Lime Karna	*Citrus karna* Raf.	IVIA – 242
‘Rough’ Lemon	*Citrus jambhiri* Lush.	IVIA – 333
Lemon ‘Eureka’	*Citrus limon* (L.) Burm.	IVIA – 297
Lemon ‘Lisbon’	*Citrus limon* (L.) Burm.	IVIA – 214
Pummelo ‘Pink’	*Citrus maxima* (Burm.) Merr.	IVIA – 275
‘Corsican’ Citron	*Citrus medica* L.	IVIA – 567

ICVN International Citrus variety numbering; IVIA Instituto Valenciano de Investigaciones Agrarias
